# Detecting False Alarms by Analyzing Alarm-Context Information: Algorithm Development and Validation

**DOI:** 10.2196/15407

**Published:** 2020-05-20

**Authors:** Chrystinne Fernandes, Simon Miles, Carlos José Pereira Lucena

**Affiliations:** 1 Department of Informatics Pontifical Catholic University of Rio de Janeiro (PUC-Rio) Rio de Janeiro Brazil; 2 Department of Informatics King's College London London United Kingdom

**Keywords:** alarm fatigue, alarm safety, false alarms, eHealth systems, remote patient monitoring, notification, reasoning, sensors

## Abstract

**Background:**

Although alarm safety is a critical issue that needs to be addressed to improve patient care, hospitals have not given serious consideration about how their staff should be using, setting, and responding to clinical alarms. Studies have indicated that 80%-99% of alarms in hospital units are false or clinically insignificant and do not represent real danger for patients, leading caregivers to miss relevant alarms that might indicate significant harmful events. The lack of use of any intelligent filter to detect recurrent, irrelevant, and/or false alarms before alerting health providers can culminate in a complex and overwhelming scenario of sensory overload for the medical team, known as *alarm fatigue*.

**Objective:**

This paper’s main goal is to propose a solution to mitigate *alarm fatigue* by using an automatic reasoning mechanism to decide how to calculate false alarm probability (FAP) for alarms and whether to include an indication of the FAP (ie, FAP_LABEL) with a notification to be visualized by health care team members designed to help them prioritize which alerts they should respond to next.

**Methods:**

We present a new approach to cope with the *alarm fatigue* problem that uses an automatic reasoner to decide how to notify caregivers with an indication of FAP. Our reasoning algorithm calculates FAP for alerts triggered by sensors and multiparametric monitors based on statistical analysis of false alarm indicators (FAIs) in a simulated environment of an intensive care unit (ICU), where a large number of warnings can lead to *alarm fatigue*.

**Results:**

The main contributions described are as follows: (1) a list of FAIs we defined that can be utilized and possibly extended by other researchers, (2) a novel approach to assess the probability of a false alarm using statistical analysis of multiple inputs representing alarm-context information, and (3) a reasoning algorithm that uses alarm-context information to detect false alarms in order to decide whether to notify caregivers with an indication of FAP (ie, FAP_LABEL) to avoid *alarm fatigue*.

**Conclusions:**

Experiments were conducted to demonstrate that by providing an intelligent notification system, we could decide how to identify false alarms by analyzing alarm-context information. The reasoner entity we described in this paper was able to attribute FAP values to alarms based on FAIs and to notify caregivers with a FAP_LABEL indication without compromising patient safety.

## Introduction

### Overview

In our previous work [1], we developed a software framework for remote patient monitoring with notification capabilities that were handled by the use of software agents. In the systems built through our framework, the anomaly detection process worked by triggering an alarm every time an anomaly occurred, independent of the circumstances [2,3].

However, these alerts are often false alarms that do not represent real danger for patients. In this case, the lack of use of any intelligent filter to detect an indication of false alarms before alerting health providers can culminate in a context of a sensory overload for the medical team. This context can result in alarm fatigue and compromise health providers’ attention, leading them to miss relevant alarms that might announce significant harmful events.

As a strategy to mitigate the alarm fatigue issue, we present a new approach to monitor patients by using an intelligent notification process supported by a reasoning mechanism. This mechanism associates a false alarm probability (FAP) to alarms based on their real-time context information, including (1) information about a patient’s circumstances, such as his or her repositioning in bed, and localization, which is tracked in real time using wearable devices with GPS, and (2) information about sensors, including battery charge life, the last time the patient’s skin was prepared to receive electrodes, and the last time electrodes were changed, among others.

After receiving this context information as input, the reasoner’s work begins by analyzing each alarm and calculating the FAP associated with it according to the false alarm indicators (FAIs) we defined, based on our literature review. Thus, the reasoner uses the FAP calculated for each alarm to decide whether to include an indication of false alarm probability (ie, FAP_LABEL) with a notification that can be visualized by caregivers.

This paper’s main goal is to propose a solution to mitigate alarm fatigue by using an automatic reasoning mechanism to assist caregivers in their decision-making process of choosing which alarms they should respond to next. Our specific goal is to attribute an FAP to each alert based on the context in which it has been generated, such as a patient’s condition and information about monitoring devices and sensors. We aim to determine the probability of an alarm being a false alarm in order to decide whether to include this information (ie, FAP_LABEL) with the notifications sent to caregivers.

We addressed the following research questions: (1) How can an automatic reasoning system calculate an indication of FAP for an alarm generated by sensors and monitoring devices? (2) How can we decide whether to add an FAP_LABEL to a notification that could be visualized by the health care team?

We defined the following hypotheses for our case study:

1. Hypothesis 1 (H1): Our reasoning algorithm should associate an FAP value to every alarm generated by sensors and monitoring devices in our experiments.
2. Hypothesis 2 (H2): Our reasoning algorithm should add an indication of an FAP to each alarm, upon which the reasoner should decide whether or not to notify caregivers with an indication of FAP (ie, FAP_LABEL).
3. Hypothesis 3 (H3): Patient safety should not be compromised if and when the reasoning algorithm decides to add an FAP_LABEL to the notification.

The main contributions of this work are as follows:

1. A list of the FAIs we defined that can be utilized and possibly extended by other researchers.
2. A novel approach to assess the probability of a false alarm using statistical analysis of multiple inputs representing alarm-context information.
3. A reasoning algorithm that uses alarm-context information to detect false alarms in order to decide whether to notify caregivers with an indication of FAP (ie, FAP_LABEL) to avoid alarm fatigue.

### Background and Related Work

#### Alarms and the Impact of Alarm Safety in Patient Care

Alarms are utilized to improve patient safety and quality of care by detecting changes early and requiring appropriate action. However, the medical literature contains many studies showing that up to 90% of all alarms in critical-care monitoring are false positives. The vast majority of all threshold alarms in the intensive care unit (ICU) do not have a real clinical impact on the care of the critically ill [4].

Many studies have recorded the number of alerts being triggered nowadays in ICUs during a period of time in order to analyze the impact of alarm safety in patient care as a consequence of the excessive volume of alarms. For instance, Kierra reported that during a 12-day analysis of the alarm system at The Johns Hopkins Hospital in Baltimore, USA, there was an average of 350 alerts per bed per day and that in one ICU, the average was 771 alerts per bed per day [5].

Lawless analyzed alarm soundings that occurred in an ICU during a 7-day period, recorded by ICU staff [6]. In his experiments, he categorized alarms into three types: false, significant (ie, resulted in change in therapy), or induced (ie, by staff manipulations; not significant). He showed that out of 2176 total alarm soundings, 1481 (68.06%) were false, 119 (5.47%) were significant, and 576 (26.47%) were induced. His results showed that over 94% of alarm soundings in a pediatric ICU may not be clinically important. Based on his findings, the author concluded that current monitoring systems are poor predictors of untoward events.

In addition to the excessive number of alarms in ICUs, another alarm-related problem, as presented by Sendelbach, is the high number of different alarm signals that was reducing the effectiveness of the alarms, creating confusion for staff, and was thus detrimental to patient care [7]. In 1983, up to six alarms could be associated with each patient in an ICU. By 1994, up to 33 different alarms were identified, and by 2011, this number increased to over 40 different alarm signals in an ICU [7]. There have been as many as 120 separate alarm devices in an operating room (OR) that are stand-alone, uncorrelated, and unprioritized [7].

The main problem of having so many different devices triggering alarms is that it is not feasible for nurses to identify all of them, which means that this increase has occurred despite staff having difficulty in learning all available alarm signals in their work environment. Staff from an OR were only able to identify between 10 and 15 out of the 26 alarms triggered in the room, and ICU nurses could only identify between 9 and 14 out of 23 alarms found in the ICU, which contributes to the alarm overload problem [7].

Kerr and Hayes [8] recognized that the excessive number and many diverse types of alarms were resulting in adverse consequences to patient care, including the following: (1) the reduction of the effectiveness of alarms, (2) creation of confusion and distraction for caregivers, who were having difficulties in responding to alarms, and (3) the deterioration of patient care, putting patients in a more unsafe environment.

Lastly, a third alarm-related problem we are focusing on in this paper is the excessive number of false alarms. Studies have indicated that false and/or clinically insignificant alarms range from 80% to 99% [9]. False alarms are frequently triggered by erroneous or absent patient data. These types of alarms can be caused by events such as patient movement or repositioning in bed and by poor placement of sensors, such as an external fetal heart rate monitor or pulse oximeter [10].

Along with the already-mentioned alarm-related problems that can affect patient care, there is more information in ICUs that is considered critical for the health care team, such as (1) the perceived alarm urgency, and (2) the perceived true alarm rate of the alarm system [10]. Tanner showed that perceived alarm urgency contributes to the nurses’ alarm response; however, nurses also use additional strategies to determine response, including the criticality of the patient, signal duration, uncommonness of the alarming device, and workload [10].

Regarding the perceived true alarm rate of the alarm system, an important finding by Tanner is the link between the impact of the perceived true alarm rate of the alarm system by caregivers and its influence on patient care. The author showed that the nurses’ responses to alarms follow the perceived true alarm rate of the alarm system. According to the author, if the true alarm rate is perceived to be 10% reliable, then the response rate will be about 10% [10].

Although alarm safety is a critical issue that needs to be addressed to improve patient care, hospitals have not given serious consideration to how their staff should be using, setting, and responding to clinical alarms, according to the Emergency Care Research Institute (ECRI) [11]. Currently, this complex and overwhelming scenario is still a problem that culminated in an unsolved health problem known as *alarm fatigue*, which we next describe.

#### Alarm Fatigue

By definition, *alarm fatigue* consists of the lack of response due to excessive numbers of alarms in hospital environments, especially in ICUs, resulting in sensory overload and desensitization [9]. This issue has the potential to compromise patient safety [12], since frequent alarms are distracting and interfere with a clinician’s performance of critical tasks. Excessive false positive alarms may lead to apathy, resulting in a lower likelihood that real events may be acted on. For their part, insignificant alarms may result in distraction and could lead to the disabling of alarm systems by staff [9].

To illustrate this scenario, studies have indicated that false and/or clinically insignificant alarms range from 80% to 99% [9]. The presence of medical devices generate enough false alarms to cause a reduction in responses, leading to a scenario in which caregivers disable, silence, and/or ignore the alarms [12] or are slow to respond [8,9].

In Table 1, we summarized the information we presented about alarm-related issues as well as their causes, consequences to the staff, consequences to patients’ care, and avoidance strategies [9].

**Table 1:** Summary of alarm-related issues.

Alarm-related issue	Causes	Consequences to the staff	Consequences to patient care	Avoidance strategies
Excessive false positive alarms	Can be attributed to patient manipulation (ie, motion artifact)	Apathy and desensitization Mistrust	Reduction in responding Lack of caregiver response Real events being less likely to be acted on	Suspension of alarms for a short period prior to patient manipulation Statistical methods should be suitable to decrease the number of false positive alarms
Frequent insignificant or irrelevant alarms	Use of the default alarm settings Poor staff education on alarm management	Distraction Reduction in trust	Disruption of patient care Disabling of alarm systems by staff	Eliminating nonessential alarms Adjusting alarm parameters on monitors to suit patients’ conditions Staff education on alarm management

#### Statistical and Artificial Intelligence-Related Approaches

According to Imhoff et al, the quality of medical device alarms is unsatisfactory, affecting quality of care and patient safety. Since the low quality of alarm-generating algorithms is one of the main causes of this problem, major improvements in alarm algorithms are urgently needed [4].

To achieve this goal, a variety of alarm-suppression algorithms have been developed and successfully applied in the laboratory and the clinical environment to avoid alarm fatigue, such as relevance vector machine learning, statistical metrics, time series analysis, spectral regression, feature selection, and other classifiers [13]. Imhoff et al showed different methods that have been proposed for use in the alarm systems of medical devices, including statistical approaches, such as improved data preprocessing, robust signal extraction, segmentation, median filter, statistical process control, and time series analysis for pattern detection, among others. Artificial intelligence (AI) methods have also been investigated and include approaches based on machine learning, neural networks, random forests, fuzzy logic, and Bayesian networks [4].

Another strategy to avoid alarm fatigue is to use notification delays that are performed through the use of a middleware between the alarming medical device and the clinicians’ receiver device, such as a mobile phone or a tablet. Several studies found that introducing alarm delays before notifying caregivers could decrease *false alarms* by 25%-67% [13]. Regarding the reduction of the total alarms, considering the effects of these interventions, alarm quantities decreased between 18.5% and as much as 89%, according to Winters et al. Fernandes et al also present a reasoning algorithm that works through the use of a notification delay strategy to mitigate alarm fatigue [14]. Other examples of promising proposed approaches are the application of contextuality and the integration of alarms to create smart alarms with improved data presentation through human factors engineering [13].

According to Imhoff et al, one of the main areas in which alarms can be improved is alarm validation (ie, determining whether the alarm is actually valid) [4]. In this work, our main contribution is to this area. Our methodological approach to deal with alarm validation involves trying to fill the gap of having feasible solutions for mitigating the alarm fatigue problem by focusing on the issue of false positive alarms, which is known to be a serious problem that still remains unsolved.

## Methods

### Overview

With regard to methodology, we present a new approach to mitigate the alarm fatigue issue. We developed an application that attributes an FAP to alarms based on FAIs that we defined. Our reasoning algorithm uses the calculated FAP to decide whether to include an indication of FAP with a notification (ie, FAP_LABEL) before sending it to caregivers, in order to assist them in the complex task of choosing the next alarms to which they should respond.

### Reasoning Model for Deciding Whether to Include an FAP Label With a Notification

In our system, a notification is a type of message that is sent to caregivers and contains information about a detected alarm or a group of alarms. An FAP is associated with an individual alarm; we calculate the FAP according to the FAIs we describe next, while an FAP_LABEL, on the other hand, corresponds to the probability of a notification containing a false alarm.

We calculate the FAP of every alarm triggered by our system. However, the reasoning algorithm decides whether to include the indication of the FAP with a notification—as the FAP_LABEL—based on the FAIs. The FAP_LABEL is the piece of information that can be visualized by caregivers. The inputs for our algorithm are a notification and its context information, including information about the patient’s conditions and sensors. After receiving these inputs, the reasoner starts working by analyzing the notification content and calculating the FAP_LABEL associated with it.

**Figure 1:**
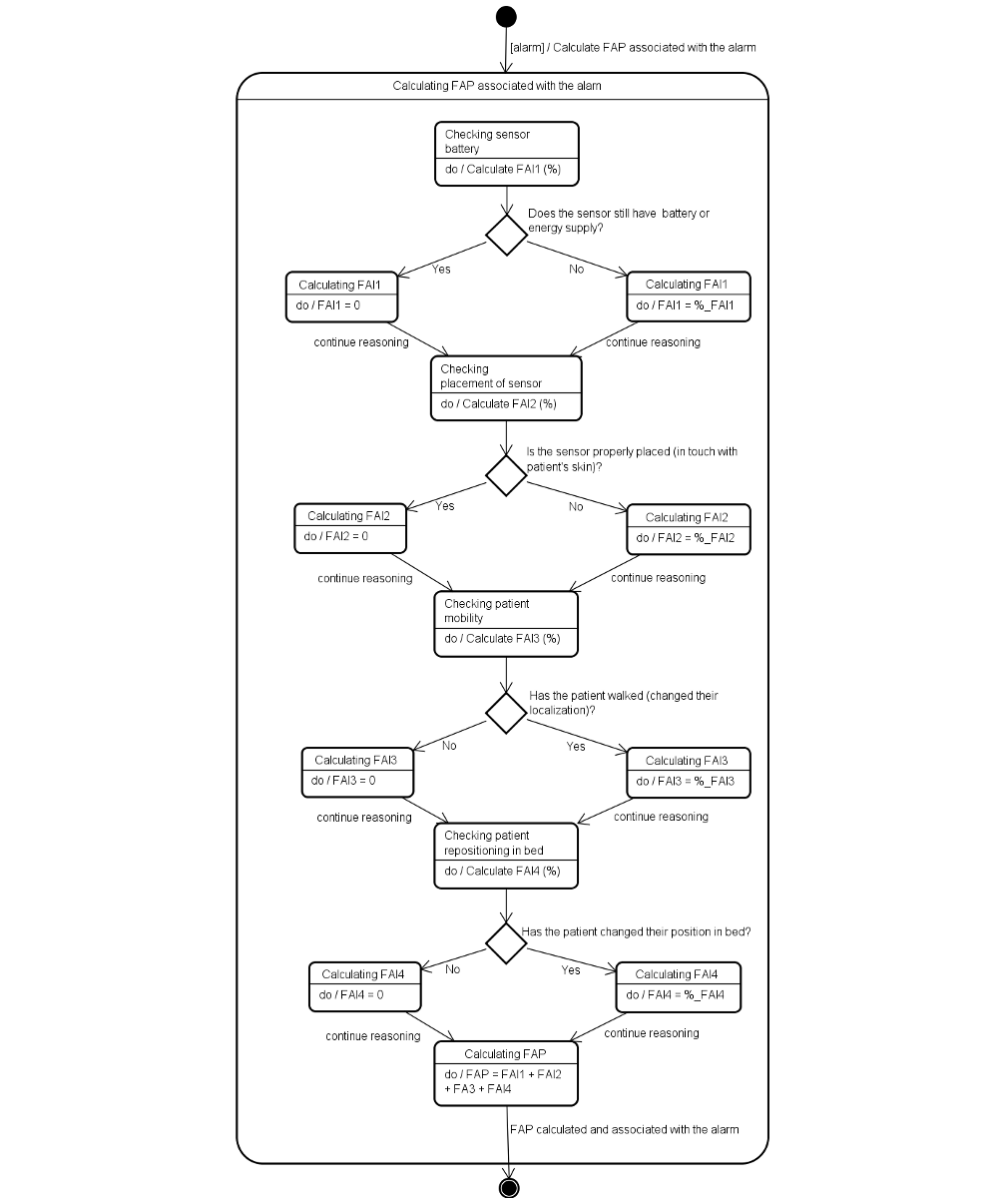
State machine diagram showing how we calculate the false alarm probability (FAP) associated with an alarm. FAI: false alarm indicator.

The processes to calculate the FAP and the FAP_LABEL are described below. Figure 1 presents a state machine diagram of the FAP reasoning process considering each alarm individually, as well as the reasoning modelling process that decides whether to notify caregivers through an FAP_LABEL indication.

### Calculation of FAP Based on the FAIs

To calculate the FAP associated with each alarm, we defined four indicators of false alarms based on the information we gathered in our literature review. According to Kerr and Hayes, the main events that cause false alarms are patient movement or repositioning in bed and poor placement of sensors. Another common issue that triggers alarms is related to technical problems, such as lack of a battery in the monitoring devices [8].

The four FAIs defined in this case study represent information about (1) the duration of a sensor battery and the last time it was changed, (2) the last time the patient’s skin was prepared to receive electrodes and the last time they were changed, (3) the patient’s mobility, and (4) the patient’s position in bed. To calculate the FAI percentage in our experiments, we considered each indicator to have the same weight. The FAIs are listed below:

1. FAI1: Sensor battery FAI (SENSOR_BATTERY_FAI). This is an indication of the FAP associated with the battery-charge level of the sensors attached to the patient.
2. FAI2: Placement of sensor FAI (PLACEMENT_OF_SENSOR_FAI). FAI2 is related to the placement of a sensor (ie, whether a sensor is properly in touch with the patient’s skin).
3. FAI3: Patient mobility FAI (PATIENT_MOBILITY_FAI). This indicator is related to patient mobility, which means that it can evaluate the probability that the alarm has been triggered due to his or her movement from the bed to other places.
4. FAI4: Patient repositioning FAI (PATIENT_REPOSITIONING_FAI). This indicator can be used to calculate the FAP related to patient repositioning (ie, whether the alarm has been sent simply because the patient may have changed his or her position in bed).

### Inputs for Our Reasoning Algorithm Regarding Whether to Add an FAP_LABEL

As shown in Table 2, we defined eight inputs for our algorithm. There are four types of information that need to be manually inserted into our system by caregivers (Inputs 1-4), two types of data automatically collected via sensors (Inputs 5 and 7), and, finally, two inputs (Inputs 6 and 8) that are retrieved from the database by the system as historical patient data. Every input mentioned above is related to one of the four FAIs, as described below.

**Table 2:** Inputs for our reasoning algorithm.

Input	Input name	FAI^a^ the input is used to calculate	Description	Type of related monitoring device
1	LEVEL_OF_ BATTERY	FAI1 (SENSOR_ BATTERY_FAI)	Level of battery for each monitoring device, including multiparametric monitors	Monitoring devices that use batteries
2	LAST_TIME_ BATTERY_ CHANGED	FAI1 (SENSOR_ BATTERY_FAI)	Last time the device’s battery was changed	Monitoring devices that use batteries
3	LAST_TIME_ SKIN_ PREPARATION	FAI2 (PLACEMENT_OF_SENSOR_FAI)	Last time skin preparation occurred	Sensors that use electrodes
4	LAST_TIME_ ELECTRODES_ CHANGED	FAI2 (PLACEMENT_OF_SENSOR_FAI)	Last time electrodes were changed	Sensors that use electrodes
5	CURRENT_ PATIENT_ LOCALIZATION	FAI3 (PATIENT_ MOBILITY_FAI)	The current patient’s localization	Sensors used to track patient localization
6	LOG_LAST_ PATIENT_ LOCALIZATION	FAI3 (PATIENT_ MOBILITY_FAI)	A log of the patient’s last localization	Sensors used to track patient localization
7	CURRENT_ PATIENT_POSITION_IN_BED	FAI4 (PATIENT_REPOSITIONING_FAI)	The current position a patient occupies in a bed	Sensors used to track patient position in bed
8	LOG_LAST_ PATIENT_POSITIONS_IN_BED	FAI4 (PATIENT_REPOSITIONING_FAI)	The last positions a patient has occupied in a bed	Sensors used to track patient position in bed

^a^FAI: false alarm indicator.

### Output of Our Reasoning Algorithm

There is one output of our algorithm—Output1: The probability that an alarm is false (ie, the FAP).

### Application’s Details: Technologies Utilized, Scenario, and Settings

To test our reasoning algorithm, we developed a system comprising an application (ie, the Producer App) that sends alarms to a broker who routes them to consumer applications that receive these alarms on behalf of the health care team. The system was developed in the Java language using the RabbitMQ message broker (Pivotal) [15]. The reason we decided to use RabbitMQ to handle the features related to data safety and scalability is to allow us to focus mainly on our functional requirements, since we are dealing with a high volume of alarms in our system.

### Application Scenario

**Figure 2:**
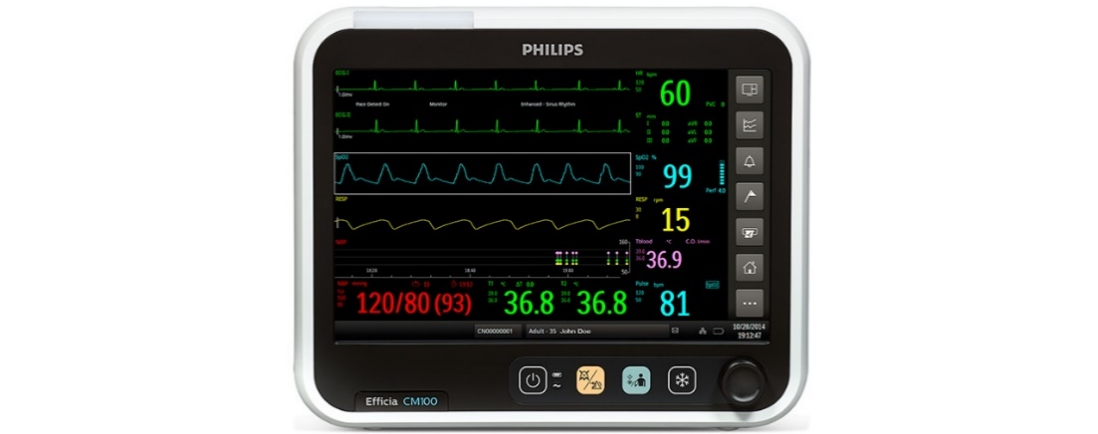
The Philips Efficia CM100 monitor.

**Figure 3:**
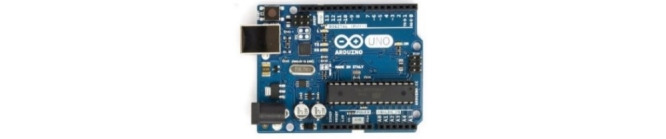
Arduino UNO microcontroller.

The application scenario consisted of a group of four patients being monitored in an ICU with sensors and monitoring devices, such as multiparametric monitors (see Figure 2), wearable devices, and external sensors that can be utilized with microcontrollers (see Figure 3).

### Monitoring Devices Used to Collect Biometric Patient Data

#### Philips Efficia CM100 Monitor

The Philips Efficia CM100 monitor [16] is commonly utilized to collect vital signs, such as electrocardiogram (ECG), breathing, temperature, noninvasive blood pressure (NIBP), oximetry (ie, peripheral oxygen saturation [SpO_2_]), capnography (ie, end-tidal carbon dioxide [EtCO_2_]), and invasive blood pressure (IBP).

#### eHealth Sensor Platform Kit

**Figure 4:**
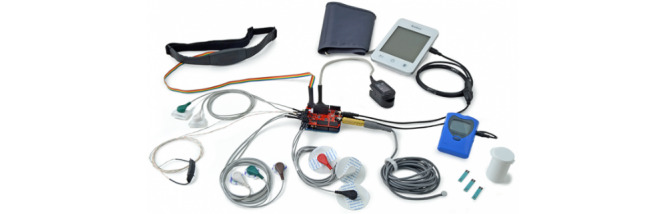
eHealth Sensor Platform Complete Kit.

**Figure 5:**
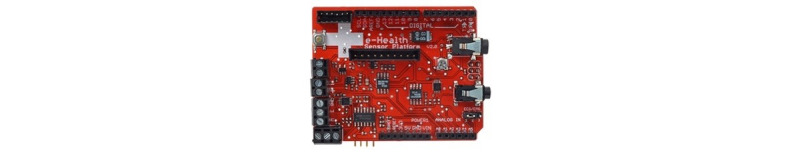
eHealth Sensor Shield.

The electronic health (eHealth) Sensor Platform Complete Kit, version 2.0 (Cooking Hacks) [17] (see Figure 4), contains an eHealth Sensor Shield (Cooking Hacks; see Figure 5) compatible with the Arduino UNO (see Figure 3) [18] and Raspberry Pi (Raspberry Pi Foundation) [19] microcontrollers. It also contains 10 sensors to collect biometric data (see Figure 4): pulse, oxygen in blood, airflow (ie, breathing), body temperature, ECG, glucometer, galvanic skin response, blood pressure, patient position (ie, accelerometer), and muscle (ie, electromyography [EMG]).

### Application Settings

In our simulated environment, patients were monitored through the use of two sensors: heart rate and temperature. The sensor readings were generated by a vital signs simulator that we developed. Regarding the sensor data simulated for each sensor, the temperature readings were generated randomly by the simulator within the 35.0°C-42.0°C range and the heart rate readings were randomly selected from the 40-188 beats-per-minute range. To define when a given temperature and heart rate reading represented an anomalous value that should trigger an alarm, we defined the thresholds shown in Table 3 for each patient.

**Table 3:** Defining the anomaly thresholds of temperature and heart rate sensors for each patient.

Patient ID	Minimum temperature, °C	Maximum temperature, °C	Minimum heart rate, BPM^a^	Maximum heart rate, BPM
1	35.5	39.0	60	100
2	35.0	38.5	55	95
3	35.5	39.5	60	100
4	35.5	38.5	50	100

^a^BPM: beats per minute.

In our experiments, we set the FAP_NOT_MIN at 75% (ie, the minimum value used as a reference to decide whether to add the FAP_LABEL to the notification). This means that every time the calculated FAP for an alarm was higher than or equal to 75%, our reasoner added the FAP_LABEL to the notification. Otherwise, we set the FAP_LABEL in our dataset to UNDEFINED, meaning that it was not included in the notification as an additional piece of information for caregivers (see Tables 4 and 5). We chose to use this strategy because we believe that only if this value is significant will it be useful to send the false alarm indication to the caregivers. Since we are working with an experimental version of our system, the choice of 75% for the FAP_NOT_MIN was selected arbitrarily. However, it is important to say that the medical staff can configure this value according to their preferences.

## Results

In Tables 4 and 5 we present the results from our experiments. We illustrate a part of the output of our reasoning algorithm showing the first 10 notifications related to the temperature and heart rate vital signs, respectively. As one can see, FAP values were attributed to the alarms, and the FAP_LABELs were added to notifications by the reasoner. The first four columns represent the Notification ID (NID), Ward ID (WID), Patient ID (PID), and Alarm ID (AID), respectively.

**Table 4:** Results of our experiments for notifications related to temperature alarms.

NID^a^	WID^b^	PID^c^	AID^d^	Sensor value, °C	Alarm timestamp, date and time	FAP^e^,%	Notification timestamp, date and time	FAP_LABEL, %
1	1	1	1	35.0	2019-07-02 21:51:06.291	50.0	2019-07-02 21:51:06.334	UNDEFINED
2	1	4	2	42.0	2019-07-02 21:51:08.328	25.0	2019-07-02 21:51:08.328	UNDEFINED
3	1	3	4	41.0	2019-07-02 21:51:12.457	50.0	2019-07-02 21:51:12.457	UNDEFINED
4	1	2	9	41.0	2019-07-02 21:51:43.223	75.0	2019-07-02 21:51:43.223	75.0
5	1	1	12	42.0	2019-07-02 21:52:03.697	50.0	2019-07-02 21:56:06.334	UNDEFINED
5	1	1	15	42.0	2019-07-02 21:52:20.053	100.0	2019-07-02 21:56:06.334	100.0
5	1	1	16	41.0	2019-07-02 21:52:24.135	75.0	2019-07-02 21:56:06.334	75.0
5	1	1	17	35.0	2019-07-02 21:52:32.309	25.0	2019-07-02 21:56:06.334	UNDEFINED
5	1	1	18	42.0	2019-07-02 21:52:42.594	50.0	2019-07-02 21:56:06.334	UNDEFINED
5	1	1	20	41.0	2019-07-02 21:52:50.774	50.0	2019-07-02 21:56:06.334	UNDEFINED

^a^NID: Notification ID.

^b^WID: Ward ID.

^c^PID: Patient ID.

^d^AID: Alarm ID.

^e^FAP: false alarm probability.

**Table 5:** Results of our experiments for notifications related to heart rate vital signs.

NID^a^	WID^b^	PID^c^	AID^d^	Sensor value, BPM^e^	Alarm timestamp, date and time	FAP^f^,%	Notification timestamp, date and time	FAP_LABEL, %
1	1	2	1	108.0	2019-07-02 21:51:09.375	75.0	2019-07-02 21:51:09.39	75.0
2	1	1	2	145.0	2019-07-02 21:51:11.432	25.0	2019-07-02 21:51:11.432	UNDEFINED
3	1	4	6	123.0	2019-07-02 21:51:21.721	50.0	2019-07-02 21:51:21.722	UNDEFINED
4	1	3	8	116.0	2019-07-02 21:51:25.827	50.0	2019-07-02 21:51:25.827	UNDEFINED
5	1	2	3	156.0	2019-07-02 21:51:15.539	0.0	2019-07-02 21:56:09.397	UNDEFINED
5	1	2	5	159.0	2019-07-02 21:51:19.667	50.0	2019-07-02 21:56:09.397	UNDEFINED
5	1	2	7	44.0	2019-07-02 21:51:23.776	75.0	2019-07-02 21:56:09.397	75.0
5	1	2	9	164.0	2019-07-02 21:51:27.874	50.0	2019-07-02 21:56:09.397	UNDEFINED
5	1	2	16	184.0	2019-07-02 21:51:44.254	25.0	2019-07-02 21:56:09.397	UNDEFINED
5	1	2	23	51.0	2019-07-02 21:52:00.641	0.0	2019-07-02 21:56:09.397	UNDEFINED

^a^NID: Notification ID.

^b^WID: Ward ID.

^c^PID: Patient ID.

^d^AID: Alarm ID.

^e^BPM: beats per minute.

^f^FAP: false alarm probability.

## Discussion

Alarm safety is a complex problem to solve, influenced by a number of factors that extrapolate technology challenges and limitations, such as human influences, difficult patient conditions, a wide variety of environmental conditions, and even staffing cultures [12]. Alarm hazards are still a big challenge for members of the health care teams in ICUs. As practice settings continue to become more technology driven, effective interventions for alarm hazards in ICU settings are crucial. Feasible strategies should be provided in order to allow nurses to respond to the call to ensure patient safety in an increasingly complex care environment [10].

In this work, we tried to fill the gap of having feasible solutions to mitigate the alarm fatigue problem by focusing on the issue of false positive alarms, known to be a serious problem that yet remains unsolved. This paper presented a reasoning algorithm to detect false alarms based on alarm-context information provided automatically by the use of sensors and wearable devices and manually by the inputs of caregivers.

In our experiments, we created a database of simulated alarm-context information to establish a basis for the development of our algorithm in order to confirm H1 and H2 in experimental settings. As we can see in the FAP column of Tables 4 and 5, every alarm generated by the sensors and monitoring devices in our experiments had an FAP value associated with it by our reasoning algorithm. Our algorithm also added an indication of an FAP (ie, FAP_LABEL) to the notifications sent to caregivers. This information is available in the FAP_LABEL column of our dataset (see Tables 4 and 5).

Regarding H3, which declares that patient safety will not be compromised if and when the reasoning algorithm decides to add an FAP_LABEL to the notification, we can assume that is confirmed, since our algorithm does not stop an alarm from being triggered even when the calculated FAP is considered very high. We can see an example of this information in the sixth row of Table 4, where the alarm (ie, AID=15) still triggered a notification (ie, NID=5), even though it had a calculated FAP of 100%.

As future work, we are planning to evolve our solution to support an optimized version of our reasoning algorithm that calculates the optimal FAP_NOT_MIN based on the real-time volume of alarms being triggered in an ICU.

Another plan for future work is to develop a machine learning–based algorithm capable of predicting both the FAP and FAP_LABEL based on a dataset that contains the ICU information history, such as patients’ conditions, sensors, and alarms.

## Abbreviations

AI: artificial intelligence
AID: Alarm ID
CNPq: National Council of Research Development
ECG: electrocardiogram
ECRI: Emergency Care Research Institute
eHealth: electronic health
EMG: electromyography
EtCO2: end-tidal carbon dioxide
FAI: false alarm indicator
FAP: false alarm probability
FAPERJ: Foundation for Research Support of the State of Rio de Janeiro
H1: Hypothesis 1
H2: Hypothesis 2
H3: Hypothesis 3
IBP: invasive blood pressure
ICU: intensive care unit
NIBP: noninvasive blood pressure
NID: Notification ID
OR: operating room
PID: Patient ID
SpO2: peripheral oxygen saturation
WID: Ward ID
